# A new cobalt(II) complex with 5-(4-pyrid­yl)tetra­zole ligands

**DOI:** 10.1107/S1600536808034880

**Published:** 2008-10-31

**Authors:** Wei-Feng Zhu, Xing-Fen Zhou

**Affiliations:** a Department of Chemistry, Nanjing Institute of Railway Technology, Suzhou 215137, People’s Republic of China; b Suzhou Institute of Trade & Commerce, Suzhou 215009, People’s Republic of China

## Abstract

A new mononuclear cobalt(II) complex, tetra­aqua­bis[5-(4-pyrid­yl)tetra­zolido-κ*N* ^5^]cobalt(II) dihydrate, [Co(C_6_H_4_N_5_)_2_(H_2_O)_4_]·2H_2_O, has been synthesized and structurally characterized. The Co^II^ atom is coordinated by two N atoms from 5-(4-pyrid­yl)tetra­zole ligands (*L*), as well as four O atoms from coordinated water mol­ecules. The mol­ecule is centrosymmetric, with pairs of equivalent ligands lying *trans* to each other in a slightly distorted octa­hedral coordination geometry. A prominent feature of the complex is the formation of a three-dimensional supra­molecular network *via* O—H⋯O and O—H⋯N hydrogen bonds.

## Related literature

The corresponding complex with manganese(II) ion as the central metal atom (Lin *et al.*, 2005 ▶) has a similar structure to that of the title complex. For related literature, see: Detert & Schollmeier (1999 ▶).
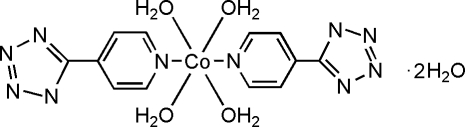


## Experimental

### 

#### Crystal data

[Co(C_6_H_4_N_5_)_2_(H_2_O)_4_]·2H_2_O
*M* *_r_* = 459.31
Triclinic, 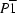

*a* = 7.2087 (16) Å
*b* = 7.8002 (17) Å
*c* = 8.6702 (18) Å
α = 91.406 (3)°
β = 90.482 (3)°
γ = 100.953 (3)°
*V* = 478.45 (18) Å^3^
*Z* = 1
Mo *K*α radiation
μ = 0.95 mm^−1^
*T* = 294 (2) K
0.20 × 0.20 × 0.14 mm

#### Data collection

Bruker SMART CCD area-detector diffractometer
Absorption correction: multi-scan (*SADABS*; Sheldrick 1996 ▶) *T*_min_ = 0.763, *T*_max_ = 0.890
2456 measured reflections
1684 independent reflections
1562 reflections with *I* > 2σ(*I*)
*R*_int_ = 0.013

#### Refinement

*R*[*F* ^2^ > 2σ(*F* ^2^)] = 0.031
*wR*(*F* ^2^) = 0.083
*S* = 1.12
1684 reflections
157 parameters
9 restraints
H atoms treated by a mixture of independent and constrained refinement
Δρ_max_ = 0.49 e Å^−3^
Δρ_min_ = −0.28 e Å^−3^

### 

Data collection: *SMART* (Bruker, 1998 ▶); cell refinement: *SAINT* (Bruker, 1998 ▶); data reduction: *SAINT*; program(s) used to solve structure: *SHELXTL* (Sheldrick, 2008 ▶); program(s) used to refine structure: *SHELXTL*; molecular graphics: *SHELXTL*; software used to prepare material for publication: *SHELXTL*.

## Supplementary Material

Crystal structure: contains datablocks I, global. DOI: 10.1107/S1600536808034880/br2083sup1.cif

Structure factors: contains datablocks I. DOI: 10.1107/S1600536808034880/br2083Isup2.hkl

Additional supplementary materials:  crystallographic information; 3D view; checkCIF report

## Figures and Tables

**Table 1:** Hydrogen-bond geometry (Å, °)

*D*—H⋯*A*	*D*—H	H⋯*A*	*D*⋯*A*	*D*—H⋯*A*
O1—H1*A*⋯N2^i^	0.855 (10)	1.968 (10)	2.795 (3)	161.84 (3)
O1—H1*B*⋯O3^ii^	0.857 (10)	1.93 (1)	2.753 (3)	161.83 (3)
O2—H2*A*⋯N3^iii^	0.849 (10)	2.10 (1)	2.939 (3)	170.66 (3)
O2—H2*B*⋯O3^iv^	0.851 (3)	1.90 (1)	2.745 (3)	172.44 (3)
O3—H3*A*⋯N5^v^	0.853 (10)	1.99 (1)	2.840 (3)	177.52 (3)
O3—H3*B*⋯N4^vi^	0.849 (10)	1.942 (10)	2.780 (3)	168.93 (3)

Symmetry codes: (i) 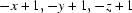; (ii) 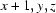; (iii) 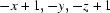; (iv) 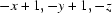; (v) 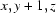; (vi) 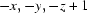.

**Fig. 1.**
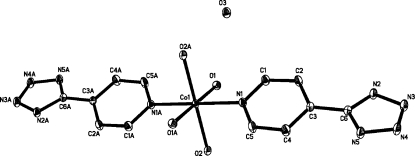
An ORTEP view of the title complex with 30% thermal ellipsoids. The H atoms are omitted for clarity.

**Fig. 2.**
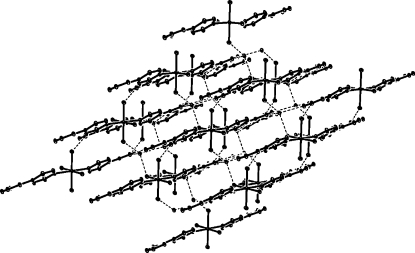
The packing diagram of the title complex.

## supplementary crystallographic information

### Comment

A wide range of applications of tetrazoles have found in areas as diverse as coordination chemistry, medicinal chemistry and materials science. The study of complexes containing substituted tetrazole ligands is of interest to describe the ways in which tetrazoles bind to metal centres. However, in the title complex reported here, the substituted tetrazole ligands bind to metal centres by the N atoms from pyridyls. In this contribution, we chose 5-(4'-pyridyl)tetrazole (*L*) as organic ligands and synthesized a new mononuclear complex, namely (Co*L*_2_(H_2_O)_4_).H_2_O.

Single crystal X-ray diffraction analysis reveals that the vicinity of the cobalt(II) ion is constituted by two nitrogen atoms from the *L* ligands and four oxygen atoms from coordinated water moleculars. The molecule is centrosymmetric, so pairs of equivalent ligands lie *trans* to each other in a slightly distorted octahedral coordination geometry. Four oxygen atoms from coordinated water moleculars occupy the four equatorial positions while two nitrogen atoms, N(1) and N(1 A) from the *L* ligands are in the axial sites. A diagram of the molecule is shown in Fig. 1. The complex is further connected into a three dimensional supramolecular structure *via* O—H···O and O—H···N hydrogen bonds, which is shown in Fig. 2.

### Experimental

The synthesis of the *L* ligand [*L* =5-(4'-pyridyl)tetrazole] was according to the previously published literature (Detert *et al.*, 1999). A solution of CoCl_2_.(H_2_O)_6_ (0.0238 g, 0.1 mmol) in 10 ml water was added to the solution of *L* (0.0310 g, 0.2 mmol) in 5 ml water, the mixture was heated with stirring. After 3 h, the mixture was cooled to room temperature and filtered. The filtrate was allowed to stand in air at room temperature for several days. Red crystals suitable for X-ray diffraction were obtained. Calculated for C_12_H_20_CoN_10_O_6_: C 31.35, H 4.35, O 15.65, Co 12.83, N 30.48%; found: C 31.32, H 4.40%.

### Refinement

To obtain a better refinement result, eight atoms, namely C(1), H(1), C(2), H(2), C(4), H(4), C(5) and H(5) have been restrained, and all the distance of C—H were fixed at 0.93 Å, with *U*_iso_(H) = 1.19*U*_eq_(C). Other hydrogen atoms were positioned geometrically and refined using a riding model.

### Crystal data

**Table:**

[Co(C_6_H_4_N_5_)_2_(H_2_O)_4_]·2H_2_O	*Z* = 1
*M**_r_* = 459.31	*F*(000) = 237
Triclinic, *P*1	*D*_x_ = 1.594 Mg m^−^^3^
Hall symbol: -P 1	Mo *K*α radiation, λ = 0.71073 Å
*a* = 7.2087 (16) Å	Cell parameters from 1859 reflections
*b* = 7.8002 (17) Å	θ = 2.7–26.3°
*c* = 8.6702 (18) Å	µ = 0.95 mm^−^^1^
α = 91.406 (3)°	*T* = 294 K
β = 90.482 (3)°	Block, red
γ = 100.953 (3)°	0.20 × 0.20 × 0.14 mm
*V* = 478.45 (18) Å^3^	

### Data collection

**Table:**

Bruker SMART CCD area-detector diffractometer	1684 independent reflections
Radiation source: fine-focus sealed tube	1562 reflections with *I* > 2σ(*I*)
graphite	*R*_int_ = 0.013
phi and ω scans	θ_max_ = 25.0°, θ_min_ = 2.4°
Absorption correction: multi-scan (SADABS; Sheldrick 1996)	*h* = −5→8
*T*_min_ = 0.763, *T*_max_ = 0.890	*k* = −8→9
2456 measured reflections	*l* = −10→7

### Refinement

**Table:**

Refinement on *F*^2^	Primary atom site location: structure-invariant direct methods
Least-squares matrix: full	Secondary atom site location: difference Fourier map
*R*[*F*^2^ > 2σ(*F*^2^)] = 0.031	Hydrogen site location: inferred from neighbouring sites
*wR*(*F*^2^) = 0.083	H atoms treated by a mixture of independent and constrained refinement
*S* = 1.12	*w* = 1/[σ^2^(*F*_o_^2^) + (0.035*P*)^2^ + 2.1646*P*] where *P* = (*F*_o_^2^ + 2*F*_c_^2^)/3
1684 reflections	(Δ/σ)_max_ < 0.001
157 parameters	Δρ_max_ = 0.49 e Å^−^^3^
9 restraints	Δρ_min_ = −0.28 e Å^−^^3^

### Fractional atomic coordinates and isotropic or equivalent isotropic displacement parameters (Å^2^)

**Table:**

	*x*	*y*	*z*	*U*_iso_*/*U*_eq_	
Co1	0.5000	0.5000	0.0000	0.02432 (16)	
O1	0.6737 (2)	0.6584 (2)	0.16137 (19)	0.0330 (4)	
O2	0.7102 (3)	0.3451 (2)	−0.0278 (2)	0.0372 (4)	
N1	0.3835 (3)	0.3267 (2)	0.1789 (2)	0.0285 (4)	
N2	0.2607 (3)	0.0110 (3)	0.6942 (2)	0.0334 (5)	
N3	0.2040 (3)	−0.1406 (3)	0.7653 (2)	0.0371 (5)	
N4	0.1459 (3)	−0.2640 (3)	0.6620 (2)	0.0376 (5)	
N5	0.1617 (3)	−0.1980 (3)	0.5206 (2)	0.0343 (5)	
C1	0.3706 (4)	0.3835 (3)	0.3237 (3)	0.0353 (6)	
H1	0.3975	0.5034	0.3441	0.042*	
C2	0.3196 (4)	0.2748 (3)	0.4445 (3)	0.0372 (6)	
H2	0.3120	0.3213	0.5435	0.045*	
C3	0.2795 (3)	0.0948 (3)	0.4185 (3)	0.0265 (5)	
C4	0.2848 (5)	0.0366 (3)	0.2677 (3)	0.0443 (7)	
H4	0.2542	−0.0823	0.2433	0.053*	
C5	0.3357 (5)	0.1551 (3)	0.1530 (3)	0.0445 (7)	
H5	0.3366	0.1127	0.0519	0.053*	
C6	0.2333 (3)	−0.0290 (3)	0.5441 (3)	0.0271 (5)	
O3	0.0218 (2)	0.5988 (2)	0.25348 (19)	0.0332 (4)	
H1A	0.670 (4)	0.7610 (19)	0.197 (3)	0.044 (8)*	
H1B	0.782 (3)	0.629 (4)	0.170 (4)	0.073 (12)*	
H2A	0.736 (4)	0.276 (3)	0.040 (2)	0.043 (8)*	
H2B	0.794 (3)	0.372 (4)	−0.096 (3)	0.056 (10)*	
H3A	0.066 (4)	0.662 (3)	0.332 (2)	0.040 (8)*	
H3B	−0.018 (4)	0.4918 (15)	0.272 (3)	0.052 (9)*	

### Atomic displacement parameters (Å^2^)

**Table:**

	*U*^11^	*U*^22^	*U*^33^	*U*^12^	*U*^13^	*U*^23^
Co1	0.0335 (3)	0.0211 (2)	0.0174 (2)	0.00208 (17)	0.00210 (16)	0.00325 (16)
O1	0.0416 (10)	0.0277 (9)	0.0283 (9)	0.0039 (7)	−0.0052 (7)	−0.0036 (7)
O2	0.0474 (11)	0.0395 (10)	0.0289 (10)	0.0169 (8)	0.0099 (8)	0.0130 (8)
N1	0.0362 (10)	0.0248 (10)	0.0229 (10)	0.0014 (8)	0.0022 (8)	0.0034 (8)
N2	0.0459 (12)	0.0276 (11)	0.0247 (11)	0.0016 (9)	−0.0018 (9)	0.0068 (8)
N3	0.0489 (13)	0.0325 (11)	0.0279 (11)	0.0019 (9)	−0.0012 (9)	0.0104 (9)
N4	0.0499 (13)	0.0291 (11)	0.0313 (12)	−0.0004 (9)	−0.0018 (9)	0.0121 (9)
N5	0.0469 (12)	0.0264 (11)	0.0267 (11)	−0.0010 (9)	−0.0011 (9)	0.0078 (8)
C1	0.0497 (15)	0.0245 (12)	0.0282 (13)	−0.0019 (10)	0.0027 (11)	0.0028 (10)
C2	0.0527 (16)	0.0340 (14)	0.0217 (12)	−0.0001 (11)	0.0022 (11)	0.0016 (10)
C3	0.0282 (11)	0.0259 (12)	0.0245 (11)	0.0026 (9)	0.0013 (9)	0.0063 (9)
C4	0.077 (2)	0.0223 (13)	0.0296 (14)	−0.0022 (12)	0.0049 (13)	0.0022 (10)
C5	0.077 (2)	0.0301 (14)	0.0217 (12)	−0.0012 (13)	0.0043 (12)	0.0026 (10)
C6	0.0280 (11)	0.0275 (12)	0.0254 (11)	0.0040 (9)	0.0000 (9)	0.0057 (9)
O3	0.0422 (10)	0.0260 (9)	0.0292 (9)	0.0002 (8)	0.0012 (7)	0.0040 (7)

### Geometric parameters (Å, °)

**Table:**

Co1—O1^i^	2.0855 (16)	N3—N4	1.304 (3)
Co1—O1	2.0855 (16)	N4—N5	1.339 (3)
Co1—O2	2.1220 (17)	N5—C6	1.331 (3)
Co1—O2^i^	2.1220 (17)	C1—C2	1.372 (3)
Co1—N1	2.1520 (19)	C1—H1	0.9300
Co1—N1^i^	2.1521 (19)	C2—C3	1.391 (3)
O1—H1A	0.855 (10)	C2—H2	0.9300
O1—H1B	0.857 (10)	C3—C4	1.377 (3)
O2—H2A	0.849 (10)	C3—C6	1.471 (3)
O2—H2B	0.851 (10)	C4—C5	1.378 (4)
N1—C5	1.331 (3)	C4—H4	0.9300
N1—C1	1.331 (3)	C5—H5	0.9300
N2—C6	1.334 (3)	O3—H3A	0.853 (10)
N2—N3	1.342 (3)	O3—H3B	0.849 (10)
			
O1^i^—Co1—O2	90.42 (7)	N4—N3—N2	109.21 (19)
O1—Co1—O2	89.58 (7)	N3—N4—N5	109.94 (19)
O1^i^—Co1—O2^i^	89.58 (7)	C6—N5—N4	104.62 (19)
O1—Co1—O2^i^	90.42 (7)	N1—C1—C2	123.5 (2)
O1^i^—Co1—N1	89.50 (7)	N1—C1—H1	118.2
O1—Co1—N1	90.50 (7)	C2—C1—H1	118.2
O2—Co1—N1	87.51 (7)	C1—C2—C3	119.9 (2)
O2^i^—Co1—N1	92.49 (7)	C1—C2—H2	120.1
O1^i^—Co1—N1^i^	90.50 (7)	C3—C2—H2	120.1
O1—Co1—N1^i^	89.50 (7)	C4—C3—C2	116.5 (2)
O2—Co1—N1^i^	92.49 (7)	C4—C3—C6	121.0 (2)
O2^i^—Co1—N1^i^	87.51 (7)	C2—C3—C6	122.5 (2)
Co1—O1—H1A	130.6 (18)	C3—C4—C5	119.8 (2)
Co1—O1—H1B	112 (2)	C3—C4—H4	120.1
H1A—O1—H1B	114.2 (17)	C5—C4—H4	120.1
Co1—O2—H2A	123.1 (17)	N1—C5—C4	123.7 (2)
Co1—O2—H2B	118.7 (18)	N1—C5—H5	118.2
H2A—O2—H2B	115.9 (17)	C4—C5—H5	118.2
C5—N1—C1	116.5 (2)	N5—C6—N2	111.4 (2)
C5—N1—Co1	121.48 (16)	N5—C6—C3	123.4 (2)
C1—N1—Co1	121.77 (16)	N2—C6—C3	125.1 (2)
C6—N2—N3	104.82 (19)	H3A—O3—H3B	114.8 (16)

Symmetry codes: (i) −*x*+1, −*y*+1, −*z*.

### Hydrogen-bond geometry (Å, °)

**Table:**

*D*—H···*A*	*D*—H	H···*A*	*D*···*A*	*D*—H···*A*
O1—H1A···N2^ii^	0.86 (1)	1.97 (1)	2.795 (3)	162 (1)
O1—H1B···O3^iii^	0.86 (1)	1.93 (1)	2.753 (3)	162 (1)
O2—H2A···N3^iv^	0.85 (1)	2.10 (1)	2.939 (3)	171 (1)
O2—H2B···O3^i^	0.85 (1)	1.90 (1)	2.745 (3)	172 (1)
O3—H3A···N5^v^	0.85 (1)	1.99 (1)	2.840 (3)	178 (1)
O3—H3B···N4^vi^	0.85 (1)	1.94 (1)	2.780 (3)	169 (1)

Symmetry codes: (ii) −*x*+1, −*y*+1, −*z*+1; (iii) *x*+1, *y*, *z*; (iv) −*x*+1, −*y*, −*z*+1; (i) −*x*+1, −*y*+1, −*z*; (v) *x*, *y*+1, *z*; (vi) −*x*, −*y*, −*z*+1.
